# Measuring Airway Obstruction in Severe Asthma in Children

**DOI:** 10.3389/fped.2018.00189

**Published:** 2018-06-26

**Authors:** Claudia Calogero, Grazia Fenu, Enrico Lombardi

**Affiliations:** Pediatric Pulmonary Unit, “Anna Meyer” Pediatric University Hospital, Florence, Italy

**Keywords:** severe asthma, lung function, children, respiratory resistance, reactance

## Abstract

Lung function is an important tool in the diagnosis and monitoring of patients with asthma at all ages. Airway obstruction is a typical feature of asthma and it can be assessed with several lung function techniques. Spirometry, respiratory resistance and reactance, and lung volumes are available to measure it at different ages and in children. The assessment of a bronchodilator response is always recommended to show the reversibility of the obstruction. Poor lung function is a predictor of poor asthma outcome and a low Forced Expiratory Volume in the first second of expiration percent predicted measured with spirometry, has been shown to be associated with a higher risk of having an exacerbation during the following year independently of the presence of asthma symptoms. In severe asthma lung function assessment is used to distinguish different phenotypes, children with severe asthma have worse airflow limitation prior to administration of a bronchodilator than children with non severe asthma. Airway resistance and reactance are indirect measurements of airway obstruction and they can be measured with the forced oscillation technique, which is feasible also in non-collaborative children. This technique can be more informative in discriminating patients with asthma from healthy controls and is able to indicate a more peripheral involvement of the airways. The role of this technique in severe asthma is still debated. In conclusion lung function is useful in the clinical management of children with severe asthma.

## Introduction

Lung function measurements are very important in the diagnosis and monitoring of patients with asthma, especially those with severe asthma. Several techniques are available for children and adults to measure airway obstruction, which is a typical feature of asthma. Furthermore, tracking of lung function, from childhood to adulthood, has been shown in several studies, highlighting that reduced lung function at young age is associated with the development of chronic obstructive pulmonary disease (COPD) in adulthood (1–3). This article will cover the main pulmonary function techniques used in children to detect airway obstruction and their clinical utility in the diagnosis and management of severe asthma in children.

## Spirometry

Spirometry indices are obtained with a forced expiratory maneuver according to the technical European Respiratory Society (ERS)/American Respiratory Society (ATS) recommendations (4). Modified acceptability criteria for spirometry in preschool children (2–5 years) have also been published (5). The Forced Expiratory Volume in the first second of expiration (FEV_1_) and its ratio to Forced Vital Capacity (FEV_1_/FVC) are considered the gold standard to assess airway obstruction in adults and children (6). These indices are sufficient to grade the severity of obstruction, which is very important in the evaluation of subjects with severe asthma. Obstruction is shown by an abnormal FEV_1_/FVC (< 80%) and is defined as mild when FEV_1_ is >70% predicted, moderate 60–69%, moderately severe 50–59%, severe 35–49%, very severe < 35% (7).

Once a baseline measure is obtained, the evaluation of the reversibility of the obstruction with the assessment of the bronchodilator response (BDR) is recommended, as this is a typical feature of asthma. A positive BDR is defined by the ERS/ATS recommendations as an increase in FEV_1_ ≥12% and ≥200 mL with respect to baseline after administration of 400 mcg of salbutamol given by metered dose inhaler via a spacer (7). However, it has been described by Tse et al. that in children this cut-off can be not enough sensitive to detect airway obstruction and can lead to a misdiagnosis of asthma (8), suggesting that a lower cut-off (8%) should be used in children.

Regarding the clinical utility of spirometry, the American Academy of Asthma, Allergy and Immunology states in the list of the Choosing Wisely Initiative “do not diagnose or manage asthma without spirometry”^1^. Also, according the most recent update of the GINA Guidelines (9) the diagnosis of asthma should be made not only based on the clinical features of the patient, such as clinical history and symptoms, but also using pulmonary function testing to detect the presence of airflow limitation. Furthermore, the NICE guidelines (10) recommend to perform spirometry in children and adults with a diagnosis of asthma to show an obstructive pattern on the test. The assessment of a bronchodilator response is also recommended to show the reversibility of the obstruction. Several guidelines on asthma also recommend to perform spirometry for monitoring asthma at each visit (10) or at least after 3 or 6 months from the beginning of treatment and on a regular basis afterwards (1–2 years) (9).

Measuring lung function is also useful as a predictor of asthma exacerbations. In the GINA guidelines (9) a low value of FEV_1_ is mentioned as a predictor of poor asthma outcome. In a retrospective study in children with asthma, a low FEV_1_ percent predicted (particularly when < 60%) has been shown to be associated with a higher risk of having an exacerbation during the following year independently of the presence of symptoms (11). Also, Schifano et al. have described in a population of 894 asthmatic children a low concordance between symptoms and lung function results and a higher grade of asthma severity was given when spirometry was taken into account (12). This is very important in children especially when a poor awareness of symptoms is detected.

Spirometry is able to measure flows at low lung volumes that are considered to be more representative of the small airways such as the forced expiratory flow between 25 and 75% of FVC (FEF_25−75_), at 50% of FVC (FEF_50_,), and at 75% of FVC (FEF_75_). The role of these indices in detecting airway obstruction in clinical practice is however controversial. Some authors have shown that these indices are more sensitive than FEV_1_ in detecting peripheral obstruction [(13)], while others have shown that they do not affect the clinical decision more than FEV_1_/FVC (14). For this reason, a recent ATS Technical Statement recommends that FEF_25−75_ should not be shown in the pulmonary function report (15).

The definition of severe asthma, according to an ERS/ATS Task Force (16), is the presence of uncontrolled symptoms despite high dose of corticosteroids and includes difficult to treat asthma and asthma resistant to therapy. Lung function assessment is necessary for confirming the diagnosis of severe asthma and to establish the phenotype. From a practical point of view, spirometry is useful in confirming the diagnosis of severe asthma in children and it should be always performed with the administration of a bronchodilator to detect airway obstruction and its reversibility. The aspect of the Flow-Volume inspiratory and expiratory loops can be also useful in the differential diagnosis of other causes of obstruction, intrathoracic or extrathoracic airway obstruction (17).

Different phenotypes of severe asthma have been described (18, 19). These phenotypes change according to age and are different in children and adults (20). Assessing airway obstruction is useful to identify the phenotypes at all age (21). Airway obstruction however, can be not present or only mild in children with severe asthma especially in between exacerbations (22–24). In respect to adults with severe asthma, children tend to have better lung function, but a greater decline overtime (25). It has been recently reported that children with severe asthma have worse airflow limitation prior to administration of a bronchodilator than children with non-severe asthma, but have similar values after BD showing an increased BDR (20).

In the TENOR Study, that included also children and adolescents, the authors have shown that acute asthmatic exacerbations can have a role in the decline of lung function, as the inflammation cascade can lead to airway remodeling and a fixed non reversible bronchoconstriction (26). In children this mechanism can be different and especially when there is eosinophilic inflammation remodeling of airways is not always present (27). Contrarily, a recent study published by Ortega et al. on lung function data retrospectively collected from the DREAM and MENSA studies on mepolizumab in severe asthmatics with eosinophilic inflammation (mainly adults with a very small proportion of adolescents), shows that a higher number of exacerbations is associated with a worsening of lung function measurements over time (28). These features can identify a more severe phenotype of asthma and each exacerbation can contribute with a loss in FEV_1_ of about 50 mL/year (28). The mechanism involved is hypothesized to be a reduced elasticity of the lung as a result of airway narrowing and remodeling associated to deposition of collagen.

Lung function measurements can be used to monitor response to therapy but few data are available on severe asthma in children. Deschildre et al. published data on 1 year of real life experience on 104 children with severe asthma treated with omalizumab showing an increase of 4.9% predicted of FEV_1_ at the end of the 12 months (29). A recent longitudinal study on lung function in severe asthma conducted in Brazil on 65 severe asthmatic patients (between 6 and 18 years of age) receiving high dose of corticosteroids showed only a small improvement of airway reversibility (FEV_1_ after bronchodilator) (30).

## Body plethysmography

Lung function obtained with the body plethysmography allows to measure airway resistance and lung volumes, with particular interest to Total Lung Capacity (TLC) and Residual Volume (RV). RV is a good index of air trapping and is a marker of hyperinflation when the value is greater than 120% predicted. This measure can add information to the conventional spirometry especially in severe asthmatic patients. It has been shown that 49% of children having airflow obstruction with an FEV_1_/FVC < 80% have a significant hyperinflation (31). Sorkeness et al. in the SARP cohort, showed that children with severe asthma were more hyperinflated and air-trapped in respect to children with non severe asthma that had normal RV and TLC (32).

## Respiratory resistance and reactance

Airway resistance, which is an indirect measurement of airway obstruction, can be evaluated with the body plethysmograph, but its measurement requires the panting maneuver which is substantially infeasible in preschool children. Several techniques have been developed, such as the interrupter technique (Rint), the forced oscillation technique (FOT), and the measurement of specific airway resistance (sRaw) using the body plethysmograph, which are performed during tidal breathing. These techniques require only minimal collaboration and are feasible also in preschool children when spirometry cannot be performed or other techniques cannot be used. In addition to respiratory resistance, FOT offers the advantage of also measuring respiratory reactance, which can be thought of as the distensibility of the respiratory system. Technical recommendations for each technique are available (5, 33) and several reference equations have been published (34–36).

The evaluation of the bronchodilator response is also possible and different cut-off values have been reported in the literature (35, 37, 38). Those techniques, with particular regard to FOT has been suggested to be more sensitive in detecting airway obstruction because of no “deep inspiration” effect on bronchial tone that is required with a forced maneuver (39, 40).

In a study conducted from Heijkenskjold Rentzhog et al. (41), the authors showed that FOT measurements were more informative in discriminating patients with asthma from healthy controls. FOT measurements were able to indicate a more peripheral involvement of the airways. Similar results are also available form previous studies on BDR in FOT resistance measurements and spirometry in children (39, 42) and adults (43). IOS results compared to spirometry have been shown to be able to predict loss of control in a group of 54 children with asthma (44). Data from 252 children with mild to moderate persistent asthma, demonstrated that the area under the reactance curve measured with IOS, was able to detect changes in airway mechanics after weeks of treatment in respect to other oscillations indices or spirometry results (45). There are some data that show that sRaw measured at preschool age correlates with mild obstruction measured with low volumes (6), also in a recent study, although on a small number of asthmatic children, sRaw is a better indices of bronchoconstriction in respect to FEV1 after a challenge test (46).

The role of these techniques in severe asthma in children remains to be assessed and further studies are needed.

## Multiple breath washout (MBW)

The Multiple Breath Washout technique measures the Lung Clearance Index (LCI), which is the cumulative expired volume required to clear an inert gas from the lungs, divided by FRC or the number of times the lung volume has to be “turned over” to wash out an inert gas such as nitrogen (N_2_) or sulfur hexafluoride (SF_6_). LCI is an index of inhomogeneity of ventilation and it has been demonstrated to be able to detect early lung damage in particular in children chronic lung disease such as Cystic Fibrosis (47), however it has some limits in measuring airway obstruction. In severe asthma with severe obstruction some areas of the lung can be excluded from ventilation giving a false value of LCI that can even appear within the normal range. S_cond_ is another index measured with MBW that refers to the inhomogeneity of the conducting airways and this might be more useful in asthmatic patients. The limits of this technique are also related to the lack of extended reference equations with particular respect to the pediatric population.

## Bronchial hyperresponsiveness

Bronchial hyperresponsiveness (BHR) can be considered as the variation of the bronchial tone that can vary after exposition to different stimuli. The stimuli that can be used as provocation test can be direct (chemicals such as histamine or methacholine, or inhaled allergens) or indirect (physical exercise, mannitol, inhaled cold air or hyperventilation with dry cold air). After a brochoprovocation test a fall in FEV_1_ of 20 or 15%, depending on the test used, is considered as a cut off value. A maximal fall of FEV_1_ between 10 or more in respect to the baseline value on two consecutive measurements after a maximal effort is considered diagnostic for exercised induced bronchoconstriction (48). In children exercise testing can be very important for the assessment of asthma in the diagnostic process but also in monitoring the response to therapy, especially when exercise symptoms are reported or there is a poor perception of symptoms (9). Methacholine bronchoprovocation test is more sensitive but less specific than exercise testing in asthmatics (49).

In a prospective study Part et al, showed in 1,041 children, the CAMP Cohort, that the percent recovery index (the percent increase in FEV_1_ after bronchodilator) in response to a bronchial challenge with methacholine was able to predict an asthmatic exacerbation in the following year (50). In severe asthma, both methacholine and exercise tests can be used to asses BHR when the diagnosis is not clear in difficult cases (16). The role of BHR in the clinical evaluation of the child with severe asthma is debatable and it is not safe to evaluate when baseline FEV_1_ is low (51).

## Conclusion

In conclusion, pulmonary function testing assessing airway obstruction is important in the clinical management of children with asthma and severe asthma during diagnosis, monitoring and evaluating response to treatment.

## Author contributions

All authors listed have made a substantial, direct and intellectual contribution to the work, and approved it for publication.

### Conflict of interest statement

The authors declare that the research was conducted in the absence of any commercial or financial relationships that could be construed as a potential conflict of interest.

## Footnotes

^1^http://choosingwisely.org/wp-content/uploads/2015/02/AAAAI-Choosing-Wisely-List.pdf
